# Comparison of intelligence, weight and height in children after general anesthesia with and without perioperative desaturation in non-cardiac surgery: a historical and concurrent follow-up study

**DOI:** 10.1186/2193-1801-3-164

**Published:** 2014-03-29

**Authors:** Maliwan Oofuvong, Alan Frederick Geater, Virasakdi Chongsuvivatwong, Thavat Chanchayanon, Juthamas Worachotekamjorn, Bussarin Sriyanaluk, Boonthida Saefung, Kanjana Nuanjun

**Affiliations:** Department of Anesthesiology, Faculty of Medicine, Prince of Songkla University, Songkhla, 90112 Thailand; Epidemiology Unit, Faculty of Medicine, Prince of Songkla University, Songkhla, 90112 Thailand; Division of Child Development, Department of Pediatrics, Faculty of Medicine, Prince of Songkla University, Songkhla, 90112 Thailand

**Keywords:** Perioperative desaturation, Weight, Height, Intelligence, Pediatric anesthesia, Non-cardiac surgery

## Abstract

**Purpose:**

To determine whether perioperative desaturation (PD) in preschool children undergoing non-cardiac surgery is associated with subsequent impairment of intelligence or subsequent change in age-specific weight and height percentile.

**Method:**

A historical-concurrent follow-up study was conducted in children aged ≤ 60 months who underwent general anesthesia (GA) for non-cardiac surgery between January 2008 and December 2011 at Songklanagarind Hospital. Children who developed PD (PD group) and children who did not develop perioperative respiratory events (no-PRE group) were matched on sex, age, year of having index GA, type of surgery and choice of anesthesia. The children’s age-specific weight and height percentile and intelligence quotient (IQ) scores by Standford Binet-LM or Wechsler Intelligence Scale for Children, 3rd edition 12–60 months after GA were compared using Student’s t- test and Wilcoxon’s rank sum test. Multivariate linear regression models for standardized IQ and multivariate mixed effects linear regression models for the change of age-specific weight and height percentile from the time of index GA to the time of IQ test were performed to identify independent predictors. The coefficients and 95% confidence intervals (CI) were displayed and considered significant if the F test p-values were < 0.05.

**Results:**

Of 103 subjects in each group (PD vs no-PRE), there were no statistically significant differences in IQ (94.7 vs 98.3, p = 0.13), standardized IQ (−0.1 vs 0.1, p = 0.14) or age-specific weight percentile (38th vs 63th, p = 0.06). However, age-specific height percentile in the PD group at the time of IQ test was significantly lower (38th vs 50th, p = 0.02). In the multivariate analysis, PD was not a significant predictor for standardized IQ (coefficient: −0.06, 95% CI: −0.3, 0.19, p = 0.57), change in age-specific weight percentile (coefficient: 4.66, 95% CI: −2.63, 11.95, p = 0.21) or change in age-specific height percentile (coefficient: −1.65, 95% CI: −9.74, 6.44, p = 0.69) from the time of index GA to the time of IQ test after adjusting for family and anesthesia characteristics.

**Conclusion:**

Our study could not demonstrate any serious effect of PD on subsequent intelligence or on the change in age-specific weight and height percentile of children after non-cardiac surgery.

**Electronic supplementary material:**

The online version of this article (doi:10.1186/2193-1801-3-164) contains supplementary material, which is available to authorized users.

## Introduction

Chronic hypoxia such as occurs in children having congenital cyanotic heart disease or sleep-disordered breathing has been reported to have an impact on long-term neurodevelopment (Hovels-Gurich et al. 2008; Ballweg et al. 2007; Hovels-Gurich et al. 2006; Sananes et al. 2012; Wise-Faberowski and Loepke 2011; Tabbutt et al. 2012; Hill et al. 2006). An intermittent hypoxic episode, especially in preterm infants, is also associated with longer-term cardiorespiratory instability, poor neurodevelopmental outcome and impaired growth (Martin et al. 2011). Perioperative desaturation (PD) is a serious perioperative respiratory event (PRE) as it could lead to cardiac arrest and hypoxic ischemic brain injury. The mechanism of PD occurring in infants with normoxia is similar to the hyperoxic state from resuscitation of acutely asphyxiated infants by using 100% oxygen, which can generate excessive neurotoxic compounds and increase oxidative stress markers (Wise-Faberowski and Loepke 2011). Moreover, a few studies have reported poor neurodevelopmental outcome following exposure to certain anesthetic agents in combination with low flow cardiopulmonary bypass for complex cardiac surgery in neonates (Sananes et al. 2012; Guerra et al. 2011).

Long-term sequelae regarding intelligence outcome in preschool level and mentally intact children having non-cardiac surgery who were exposed to general anesthesia (GA) and developed PD has never been investigated. Thus, PD occurring at a young age regardless of severity combined with GA might possibly have a long-term impact on weight, height and intelligence in preschool and school children. Understanding the long-term consequence may assist the clinician to advise the parents to plan for an affected child’s education process. Therefore, the objectives of this study were to compare subsequent intelligence and change in age-specific weight and height percentile of children undergoing GA at age ≤ 60 months who developed PD and those who had no PRE when having non-cardiac surgery at a tertiary care hospital in southern Thailand.

## Materials and methods

A joint historical-concurrent follow-up study was conducted at Songklanagarind Hospital, an 853-bed tertiary care hospital in southern Thailand, after approval from the Ethics Committee, Prince of Songkla University, on November 15th, 2012 (ClinicalTrials.gov: NCT01965093). Children aged ≤ 60 months undergoing GA between 2008 and 2011 who developed intraoperative or post-anesthetic care unit (PACU) desaturation (PD group) were compared with others who did not develop any PRE (no-PRE group) in respect of subsequent intelligence and subsequent change in age-specific weight and height percentile. Children’s weight and height parameters and intelligence 12–60 months after exposure to GA were assessed between 2012 and 2013. Written informed consent was obtained from all parents who allowed their children to participate in the study.

### Participants

The anesthetic database and the anesthetic record of children aged ≤ 60 months undergoing GA between January 2008 and December 2011 were reviewed. Patients were excluded if they were classified as American Society of Anesthesiologists (ASA) classification 4 or 5, had a preoperative oxygen saturation at room air < 95%, were already endotracheally intubated and/or mechanically ventilated prior to surgery, had congenital cyanotic heart disease or cardiac surgery, had neurosurgery, had preoperative delayed development, or had perinatal hypoxia or fetal growth retardation.

### The main exposure (PD and no-PRE)

In our hospital, the anesthetic record is recorded under anesthesia surveillance using continuous pulse oximetry and capnograph monitoring incorporated with the patient’s vital signs every 5 minutes. Time of PRE (desaturation, laryngospasm, bronchospasm, upper airway obstruction, reintubation and others) occurring as well as the lowest oxygen saturation by pulse oximetry (SpO_2_) intraoperatively or at PACU are recorded immediately in the vital sign’s table and details of PRE are described in the events section. A specific code for each type of PRE is recorded in the anesthetic database. PD was defined as a condition with oxygen saturation < 95% for more than 10 seconds (Xue et al. 1996) as recorded in the anesthetic database and the anesthetic record. Children with PD were identified from the anesthetic database and confirmed by review of the anesthetic record by the primary investigator (MO).

No-PRE children were defined as those who did not develop any respiratory event intraoperatively or at the PACU period. There was a total of 4639 no-PRE children in the study period. No-PRE children who matched each PD child on sex, age at index GA (within ± 6 months-range for age < 12 months, within ± 12 months-range for age ≥ 12 months), year of having index GA, type of surgery and choice of GA were listed. The variables that did not exist in the hospital database such as maternal education, socioeconomic status, or training by parents, could not be matched. After applying exclusion criteria, only 3 to 4 children were eligible to be included in the no-PRE matched set for each PD child, and from these one no-PRE child was selected using simple random sampling. If type of surgery could not be matched exactly with a child from the matching list, another type of surgery using the same choice of GA was used for matching.

The parents of children in both groups were contacted by the primary investigator (MO) by phone call and/or invitation postcard. If a parent of the PD child declined or could not be reached, the whole matched set was dismissed. If a selected no-PRE child was not available while the PD child was, a second no-PRE child was randomly chosen from the list.

### Outcome of interest

The study subjects of both groups were called back to the study hospital to be assessed on intelligence, weight and height. The timing of IQ tests was arranged so that the age of the matched pair at the time of IQ test differed by no more than 12 months. The duration from the GA to this follow-up assessment (at the time of IQ test) varied almost uniformly between 1 and 5 years. Evaluations were done in each member of a matched pair within a 3-month interval.

Intelligence quotient (IQ), which was the main outcome, was evaluated. Two intelligence tests were employed, namely the Standford Binet form L-M (Becker 2003) and The Wechsler Intelligence Scale for Children, 3rd edition (Watkins 1996; Freberg et al. 2008). The former was used as the main intelligence outcome in our study. It measures cognitive ability regarding verbal reasoning, quantitative reasoning, abstract and visual reasoning and short-term memory skills with a wide range of IQ scores varying from 20 to 140. The suitable age for using this test is between 2 to 7 years. The Wechsler Intelligence Scale for Children, 3rd edition test was used as a supplemental test for children who reached the ceiling age of 7 years on the Standford Binet form L-M test. It consists of verbal and performance subtests (nonverbal subtest) with a range of IQ scores varying from 70 to 130. A pediatric psychometrist (TD) established rapport with each child and performed the IQ test in all children. Children aged between 6 to 8 years were screened for the appropriate test using the last page of the Standford Binet form L-M test and then either continued with the Standford Binet form L-M test or switched to the Wechsler Intelligence Scale for Children, 3rd edition, to fit their mental age. Age-normalized IQ was computed using the table in the manual to correlate the mental age from total raw scores with actual age. As these two tests contain different items and use different methods of calculation, the normalized IQ score, standardized within IQ test were calculated and used as the final intelligence variable. Therefore, the final intelligence score had mean of 0 and standard deviation (SD) of 1.

Body weight and height of children wearing light clothing with empty pockets were measured using a Detecto scale and stadiometer. The weight and height of each child were compared with the weight-to-height data from the Nutrition Division, Ministry of Public Health, to obtain age-specific weight and height percentiles. Then, weight, age-specific weight percentile (ASWP), height and age-specific height percentile (ASHP) were compared between the two groups both at the time of index GA and at the time of IQ test. Age (months) at first talk (first meaningful word) and at first walk (walk without assistance) were also recorded.

### Potential confounding variables

Child- and family-related characteristics were obtained during interview by MO while anesthesia-related variables were obtained from the anesthesia database by BS, BS and KN and review of the anesthetic recorded by MO. Child- and family-related characteristics (12 variables) included history of prematurity, history of parental smoking, religion, mother’s age at delivery, mother’s occupation, father’s occupation, monthly quintile of family income (baht), mother’s education, father’s education, children’s education, training by parents (none, partial or regular) (Additional file 1) and child’s capability (needing full help, partial help or no help) (Additional file 2) (Table 1). Variables to represent training by parents and child’s capability were constructed by the investigator team to be included as potential confounders. They were used for the research purposes only.

**Table 1:** **Comparison of demographic data and family-related variables between the two groups**

Variables	PD (n = 103)	No-PRE (n = 103)	p-value
*Demographic data*			
Sex (M:F)	66:37	66:37	-
Age at index GA (months)^+^	22 (11.5-41)	27 (10.5-41.5)	-
Weight at index GA (kg)^a,+^	10 (7.9-13.4)	11 (7.8-15.6)	0.11
ASWP at index GA^a,+^	25th (3th-63th)	38th (10th-75th)	0.007*
Height at index GA (cm)^a,+^	81 (70–95)	85 (71–100)	0.38
ASHP at index GA^a,+^	38th (5th-63th)	38th (18th-63th)	0.10
*Family-related variables*			
Parental smoking^b,ǂ^			1
No	42 (40.8)	43 (41.7)	
Yes	61 (59.2)	60 (58.3)	
Religion^b,ǂ^			0.86
Buddhist	81 (78.6)	83 (80.6)	
Muslim	22 (21.4)	20 (19.4)	
Mother‘s age at delivery (yr)^c,§^	29.7 (6.2)	30.3 (5.9)	0.44
Mother’s occupation^b,ǂ^			0.11
None	13 (12.6)	13 (12.6)	
Company employee	33 (32.0)	27 (26.2)	
Farmer	27 (26.2)	20 (19.4)	
Government officer	8 (7.8)	21 (20.4)	
Self-employee	22 (21.4)	22 (21.4)	
Father’s occupation^d,ǂ^			0.31
None	1 (1.0)	2 (1.9)	
Company employee	33 (32.0)	32 (31.1)	
Farmer	36 (35.0)	24 (23.3)	
Government officer	16 (15.5)	24 (23.3)	
Self-employee	17 (16.5)	21 (20.4)	
Income quintiles (baht)^b,ǂ^			0.16
< 10,000	23 (22.3)	27 (26.2)	
10,001-17,800	19 (18.4)	12 (11.7)	
17,801-30,000	33 (32.0)	28 (27.2)	
30,001-40,000	7 (6.8)	17 (16.5)	
40,001-500,000	21 (20.4)	19 (18.4)	
Mother’s education^b,ǂ^			0.70
Primary school	21 (20.4)	19 (18.4)	
Secondary school	35 (34.0)	31 (30.1)	
Tertiary education	47 (45.6)	53 (51.5)	
Father’s education^b,ǂ^			0.83
Primary school	23 (22.3)	25 (24.3)	
Secondary school	35 (34.0)	31 (30.1)	
Tertiary education	45 (43.7)	47 (45.6)	
*Child-related variables*			
Prematurity^b,ǂ^			0.86
No	81 (78.6)	83 (80.6)	
Yes	22 (22.4)	20 (19.4)	
Children’s education^b,ǂ^			0.17
Home	18 (17.5)	11 (10.7)	
Nursery	7 (6.8)	12 (11.7)	
Pre-kindergarden	10 (9.7)	15 (14.6)	
Kindergarden	60 (58.3)	51 (49.5)	
Primary school	8 (7.8)	14 (13.6)	
Training by parents^d,ǂ^			0.41
None	4 (3.9)	1 (1.0)	
Partial	67 (65.0)	66 (64.0)	
Regular	32 (31.1)	36 (35.0)	
Children’s capability^d,ǂ^			0.77
Full help	1 (1.0)	0 (0)	
Partial help	36 (35.0)	34 (33.0)	
No help	66 (64.0)	69 (67.0)	
First talk (months)^a,+^	18 (15–24)	18 (15–21)	0.97
First walk (months)^a,+^	14 (12–18)	14 (12–18)	0.38

^a^Rank sum test, ^b^Chi-square test, ^c^Student’s t- test, ^d^Fisher’s exact test, *p-value < 0.05, ^+^median (IQR), ^ǂ^number (%), ^§^mean (SD), Monthly income quintiles, 1 baht = US$ 0.033 at the time of data collection, ASWP; Age-specific weight percentile, ASHP; Age-specific height percentile, GA; General anesthesia, PD; Perioperative desaturation, PRE; Perioperative respiratory event, yr; years.

Eleven anesthesia-related variables were ASA classification, number of repeated GA, type of surgery, choice of GA, technique of GA, induction agent, intubating agent, inhalation agent, gas mixed with oxygen, narcotic and duration of GA (Table 2).

**Table 2:** **Comparison of anesthesia-related variables between the two groups**

Variables	PD (n = 103)	No-PRE (n = 103)	p-value
Repeated GA (per time)^a,+^	0 (0–2)	0 (0–1)	0.08
ASA classification^b,ǂ^			0.32
1	17 (16.5)	13 (12.6)	
2	67 (64.1)	77 (73.8)	
3	20 (19.4)	14 (13.6)	
Type of surgery^ǂ^			-
Abdominal	36 (34.9)	37 (35.9)	
Ear nose throat	20 (19.4)	22 (21.4)	
Eye	9 (8.7)	9 (8.7)	
Thoracic	8 (7.8)	7 (6.8)	
Orthopedic	18 (17.5)	18 (17.5)	
Cardiac catheterization	4 (3.9)	4 (3.9)	
Superficial	8 (7.8)	6 (5.8)	
Choice of GA^ǂ^			-
GA only	67 (65.0)	64 (62.1)	
GA combined RA or PNB	36 (35.0)	39 (37.9)	
Technique of GA^c,ǂ^			0.77
Mask	11 (10.7)	9 (8.7)	
Laryngeal mask airway	26 (25.2)	23 (22.3)	
Total intravenous anesthesia	1 (1.0)	1 (1.0)	
ETT with inhalation	7 (6.8)	10 (9.7)	
ETT with balanced	58 (56.3)	60 (58.3)	
Induction agents^c,ǂ^			0.46
Thiopental	55 (53.4)	55 (53.4)	
Sevoflurane	38 (36.9)	43 (41.7)	
Propofol	8 (7.8)	3 (2.9)	
Ketamine	2 (1.9)	2 (1.9)	
Intubating agents^c,ǂ^			0.24
Succinylcholine	20 (19.4)	12 (11.7)	
NDMR	43 (41.7)	53 (51.5)	
Sevoflurane	1 (1.0)	4 (3.9)	
Propofol	1 (1.0)	1 (1.0)	
None	38 (36.9)	33 (32.0)	
Inhalation agent^c,ǂ^			0.30
Sevoflurane	88 (85.4)	96 (93.2)	
Isoflurane	5 (4.9)	3 (2.9)	
Desflurane	8 (7.8)	3 (2.9)	
None	2 (1.9)	1 (1.0)	
Gas mixed with oxygen^c,ǂ^			0.49
Air	57 (55.3)	51 (48.5)	
Nitrous oxide	44 (42.7)	52 (50.5)	
100% oxygen	2 (1.9)	1 (1.0)	
Narcotics^c,ǂ^			0.41
Intravenous fentanyl	79 (76.7)	75 (72.8)	
Intravenous morphine	2 (1.9)	4 (3.9)	
Epidural morphine	14 (13.6)	11 (10.7)	
None	7 (6.8)	13 (12.6)	
Duration of GA (hours)^b,ǂ^			0.03*
< 1	10 (9.7)	21 (20.4)	
1-3	78 (75.7)	75 (72.8)	
>3	15 (14.6)	7 (6.8)	
Perioperative lowest oxygen saturation ^a,+^	91 (70–93)	99 (98–99)	0.001*

^a^Rank sum test, ^b^Chi-square test, ^c^Fisher’s exact test, *p-value < 0.05, ^+^median (IQR), ^ǂ^number (%), ASA; American Society of Anesthesiologists, ETT; Endotracheal tube intubation, GA; General anesthesia, NDMR; Non depolarizing muscle relaxant, PD; Perioperative desaturation, PNB; Peripheral nerve block, PRE; Perioperative respiratory event, RA; regional anesthesia.

### Statistical analysis

Descriptive statistics of various variables, including frequency (%), mean and SD, median and interquartile ranges (IQR) in the PD and the no-PRE groups were computed. Student’s t-test or non-parametric rank sum test as appropriate for continuous variables and Chi-square test or Fisher’s exact test as appropriate for categorical variables were employed to check for significant difference of background characteristics of the two groups and their differences in parameters of weight, height and IQ. As the two groups were not perfectly balanced, final comparison on intelligence, age-specific weight and height percentile were adjusted for potential confounders using multivariate analysis.

To examine the relationship between PD and subsequent normalized IQ score, standardized within IQ test, a multivariate linear regression model was developed. All variables with p ≤ 0.2 in univariate analysis or otherwise of interest were included in the initial model, and the model refined by sequential backward elimination of non-significant variables (likelihood ratio test). Some significant variables between the PD and no-PRE groups were forced to remain in the final model of multivariate linear regression even though they were non-significant variables in the final model. After obtaining the final model, PD was replaced with a variable indicating severity of PD (no-PRE, mild to moderate and severe PD).

To model the relationship between PD and age-specific weight and height percentile, data were reshaped into long format (412 records) to expand the age-specific weight and height percentile into 2 times: at the time of index GA and at the time of IQ test and these percentiles were considered as repeated measures to be fitted into the mixed effects model. Final comparison of change in weight and height between PD and no-PRE group was adjusted for factors significantly associated with the age-specific weight and height percentile at the 2 times using multivariate mixed effects linear regression models with random intercept by fitting an interaction term between time and main exposure (time × PD). Some significant variables between the PD and no-PRE groups were forced to remain in the final model of multivariate mixed effects linear regression even though they were non-significant variables in the final model.

The coefficients and 95% confidence intervals (CI) were displayed and considered significant if the F test p-values were < 0.05.

### Sample size calculation

For a power of 90% and type I error of 5%, 85 children per group were required to detect a difference of ≥ 0.5 SD (i.e., a clinically significant mean difference of IQ score of 6 and SD of 12), based on a study by Sungthong et al. (Sungthong et al. 2004) of southern Thai children’s IQ at age < 9 years. A total of 100 children per group would be required to compensate for 15% drop out.

## Results

**Figure 1:**
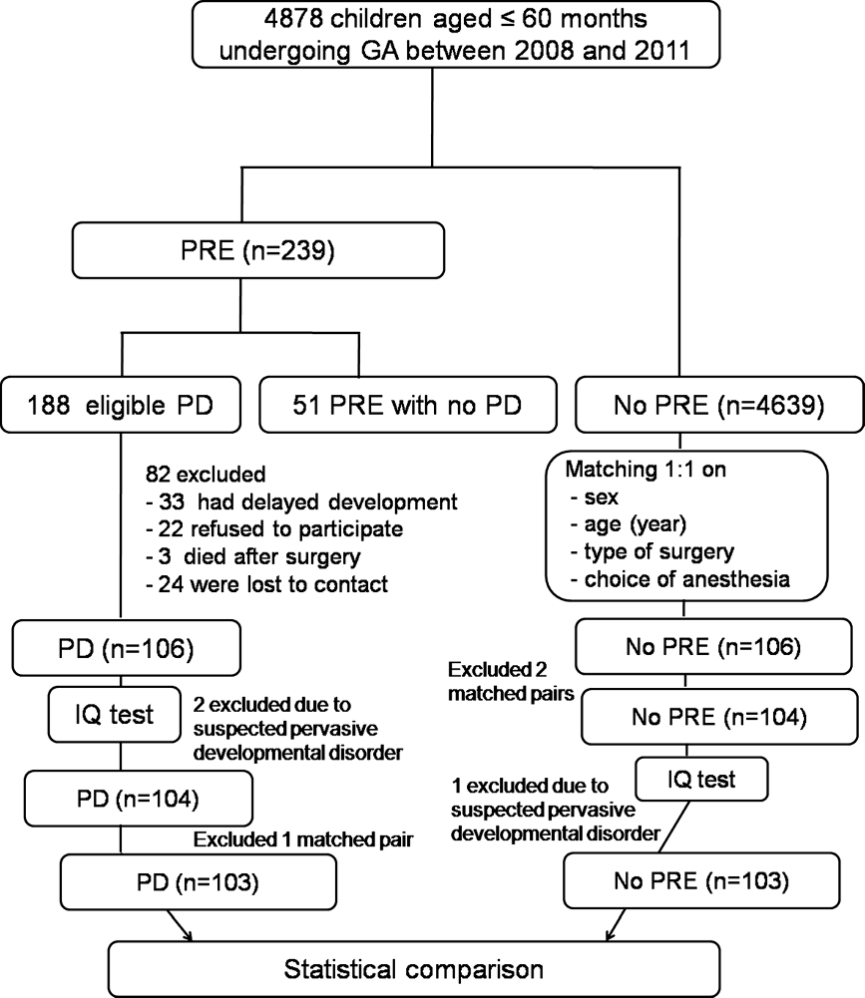
**A flow diagram of the study.** GA; General anesthesia, IQ; Intelligence quotient, PD; Perioperative desaturation, PRE; Perioperative respiratory event.

Figure 1 shows a flow diagram of our cohort study. A total of 188 children aged ≤ 60 months having PD after GA from January 2008 to December 2011 at Songklanagarind Hospital was identified. All of these children had duration of PD < 60 seconds. Eighty-five were excluded for the following reasons: 33 had preoperative delayed development, 22 refused to participate, 24 were lost to contact, 3 died after surgery and 3 matched pairs were excluded (2 PD children and 1 no-PRE child were suspected of having pervasive developmental disorders (PDD)). Among the 103 PD children, causes of PD were hypoventilation (32%), laryngospasm (26%), bronchospasm (21%), upper airway obstruction (9%), accidental extubation (5%), pulmonary aspiration (3%), difficult airway (3%) and pneumothorax (1%). Forty-one (40%) and 62 children (60%) developed severe (SpO_2_ ≤ 85%) and mild to moderate PD (SpO_2_ 86-94%), respectively. These 103 children in the PD group were matched with 103 children with no-PRE. Following the IQ test, children who were suspected of having PDD or had an IQ score < 70 were sent to the child developmental clinic.

### Association between PD and PDD

Two children from the PD group were suspected of having PDD and therefore excluded. The two no-PRE children from matched pairs of PD group having PDD were not called back at the time of assessment because they would also be excluded. In addition, one child from the no-PRE group was suspected of having PDD and this matched pair was also excluded. However, to explore the possible association between PD and PDD based on 3 possible results, a sensitivity analysis was conducted. Two-by-two tables of the 3 possible results were constructed and the Fisher’s exact test was performed to determine the association in each situation. The results showed no association between PD and PDD in any of the 3 situations: no children, 1 child or 2 children having PDD (odds ratio: 2.02, 95% CI: 0.18-22.6, p = 1, odds ratio: 1.0, 95% CI: 0.14-7.23, p = 1, odds ratio: 0.66, 95% CI: 0.11-4.03, p = 1, respectively).

Table 1 compares children’s baseline demographic data and family-related variables between the two groups. Only ASWP at index GA differed significantly between the two groups. The ASWP at index GA in the PD group was significantly lower than that in the no-PRE group (25th vs 38th, p = 0.007). Table 2 compares anesthesia-related variables between the two groups. There were no significant differences in anesthesia-related variables between the two groups except duration of GA. Duration of GA in the PD group was significantly longer than that in the no-PRE group (115 vs 85 minutes, p = 0.001). The median perioperative lowest oxygen saturation was significantly lower in the PD group than in the no-PRE group (91% vs 99%, p = 0.001).

Table 3 shows a comparison of weight, height and intelligence at the time of IQ test between the two groups. None of the outcome variables except those related to weight and height differed significantly between the two groups. The weight (kg), height (cm) and ASHP of children were significantly higher in the no-PRE group than in the PD group at the time of IQ test (18 vs 16.6 kg, 107 vs 104 cm, 50th vs 38th, p = 0.04, p = 0.01 and p = 0.02, respectively). Subgroup analysis in the PD group showed no differences in age-normalized IQ among severe (SpO_2_ ≤ 85%) and mild to moderate PD (SpO_2_ 86-94%) compared to no-PRE group (93.6, 95.5 vs 98.3, p = 0.27, respectively).

**Table 3:** **Weight, height and intelligence at the time of IQ test**

Variables	PD (n = 103)	No-PRE (n = 103)	p-value
Age (months)^+^	58 (43–72)	58 (46.5-74)	-
Weight (kg)^a,+^	16.6 (14–19)	18 (15–21)	0.04*
ASWP^a,+^	38th (18th-63th)	63th (32th-75th)	0.06
Height (cm)^a,+^	104 (97–112)	107 (101–116)	0.01*
ASHP^a,+^	38th (18th-63th)	50th (25th-75th)	0.02*
Mental age (months)^a,+^	58 (40.5-67)	60 (44.5-74)	0.08
Age-normalized IQ^b,ǂ^	94.7 (16.0)	98.3 (17.5)	0.13
Normalized IQ score, standardized within IQ test^b,ǂ^	−0.10 (0.9)	0.10 (1.0)	0.14
Intelligence test^c,§^			0.25
SB-LM	90 (87.4)	83 (80.6)	
WISC III	13 (12.6)	20 (19.4)	
Age-normalized IQ by each test			
IQ score by SB-LM^b,ǂ^	94.5 (16.5)	98.1 (18.7)	0.18
IQ score by WISC III^b,ǂ^	96.8 (12.5)	99.2 (11.7)	0.57
Age-normalized IQ by lowest			0.27
SpO_2_^d,ǂ^			
SpO_2_ ≥ 95		98.3 (17.5)	
SpO_2_ 86-94	95.5 (16.1)		
SpO_2_ ≤ 85	93.6 (16.1)		

^a^Rank sum test, ^b^Student’s t- test, ^c^Chi-square test, ^d^ANOVA test, *p-value < 0.05, ^+^median (IQR), ^ǂ^ mean (SD), ^§^number (%), ASWP; Age-specific weight percentile, ASHP; Age-specific height percentile, IQ; Intelligence quotient, PD; Perioperative desaturation, PRE; Perioperative respiratory event, SB-LM; Standford Binet form L-M, SpO_2_; Oxygen saturation by pulse oximetry, WISC III; Wechsler Intelligence Scale for Children, 3rd edition.

### Univariate and multivariate analysis of the normalized IQ score, standardized within IQ test related to PD

All 13 child- and family-related variables including ASWP at the time of index GA and 12 anesthesia-related variables including occurrence of PD were examined in the univariate analysis of normalized IQ score, standardized within IQ test. The following 8 variables having p ≤ 0.2 in the univariate analysis were included in the initial multivariate model but became non-significantly related to normalized IQ score, standardized within IQ test in the multivariate analysis: religion, mother’s age at delivery, mother’s occupation, father’s occupation, income quintiles, child’s education, children’s capability and gas mixed with oxygen. Table 4 shows results of the multivariate linear regression analysis predicting normalized IQ score, standardized within IQ test, from exposure to PD and other confounding variables. After adjusting for ASWP at the time of index GA, mother’s education, father’s education, training by parents, repeated times of GA and duration of GA, PD was not an independent risk factor for normalized IQ score, standardized within IQ test (coefficient: −0.06, 95% CI: −0.3, 0.19, p = 0.57). After replacing PD with severity of PD, there was no evidence that severity of PD was an independent risk factor for normalized IQ score, standardized within IQ test (p = 0.56) in our study.

**Table 4:** **Multivariate linear regression predictingthe normalized IQ score, standardized within IQ test from PD exposure (n = 206)**

Variables	Multivariate	
	Coefficient (95% CI)	p-value
Age-specific weight percentile at index GA	0.003 (−0.001, 0.007)	0.16
Mother’s education (ref = Tertiary education)		< 0.001
Secondary school	−0.34 (−0.67, −0.01)	
Primary school	−0.38 (−0.83, 0.06)	
Father’s education (ref = Tertiary education)		0.007
Secondary school	−0.43 (−0.75, −0.11)	
Primary school	−0.35 (−0.78, 0.07)	
Training by parents (ref = Regular)		0.007
Partial	−0.41 (−0.69, −0.13)	
None	−1.0 (−1.84, −0.15)	
Repeated GA (per time)	−0.04 (−0.08, −0.01)	0.01
Duration of GA (hours) (ref = <1)		0.37
1-3	0.07 (−0.28, 0.41)	
> 3	−0.21 (−0.7, 0.27)	
PD (ref = No-PRE)		0.57
Yes	−0.06 (−0.3, 0.19)	
Severity of PD (ref = No-PRE)		0.56
Mild to moderate PD (SpO_2_ 86-94)	0.02 (−0.27,0.31)	
Severe PD (SpO_2_ ≤ 85)	−0.16 (−0.49, 0.16)	

p-value by F test statistic, GA; General anesthesia, IQ; Intellligence quotient, PD; Perioperative desaturation, PRE; Perioperative respiratory event, SpO_2_; Oxygen saturation by pulse oximetry.

### Univariate and multivariate analysis for change in age-specific weight and height percentile related to PD

**Figure 2:**
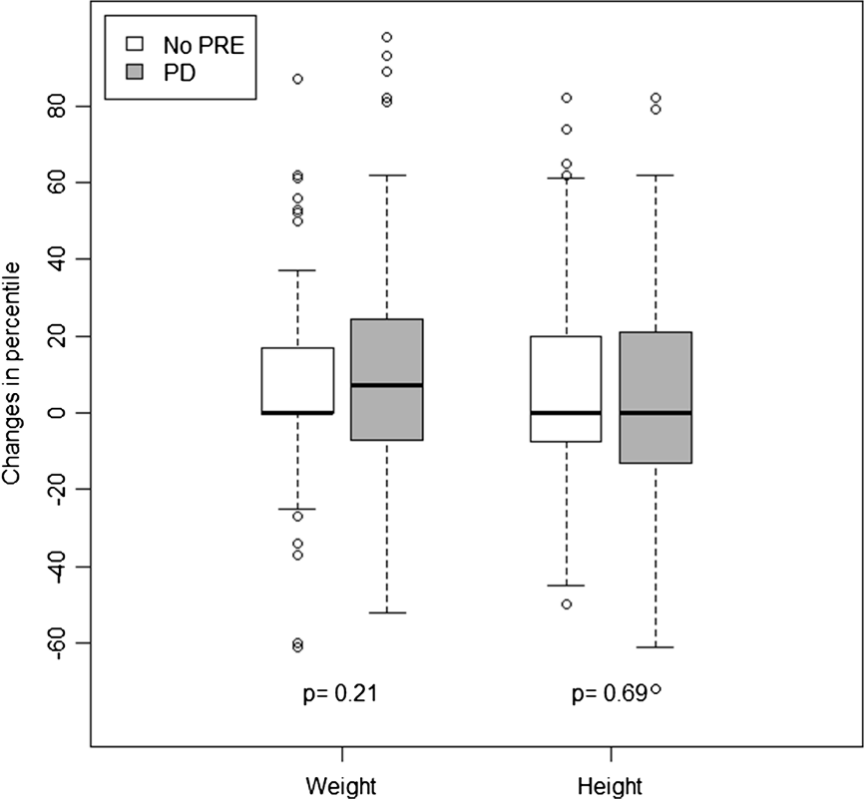
**The change of weight and height percentile from index GA to the IQ test in each group.** GA; General anesthesia, IQ; Intelligence quotient, PD; Perioperative desaturation, PRE; Perioperative respiratory event.

All 12 child- and family-related variables and 12 anesthesia-related variables including occurrence of PD were examined in the univariate analysis of the change in age-specific weight and height percentile. The following 7 variables having p ≤ 0.2 in the univariate analysis were included in the initial multivariate model but became non-significantly related to change in ASWP in the multivariate analysis: history of prematurity, religion, mother’s education, father’s education, ASA classification, type of surgery and technique of GA. The following 3 variables having p ≤ 0.2 in the univariate analysis were included and became non-significantly related to change in ASHP in the multivariate analysis: children’s capability, type of surgery and duration of GA. Table 5 shows the results of the multivariate mixed effects linear regression models predicting the change in age-specific weight and height percentile from the time of index GA to the time of IQ test after exposure to PD and other confounding variables. After adjusting for income quintile, number of times of repeated GA and duration of GA, PD was not an independent risk factor for change in ASWP (coefficient: 4.66, 95% CI: −2.63, 11.95, p = 0.21). Similarly, after adjusting for income quintile, number of times of repeated GA, history of prematurity, choice of GA , technique of GA and duration of GA, PD was not an independent risk factor for change in ASHP (coefficient: −1.65, 95% CI: −9.74, 6.44, p = 0.69). However, PD was significantly associated with a lower ASWP at the time of index GA (coefficient: −12.6, 95% CI: −21.3, −3.76, p = 0.005). Figure 2 shows the change in age-specific weight and height percentile from the time of index GA to the time of IQ test in each group.

**Table 5:** **Multivariate mixed effects linear regression predicting the change of age-specific weight and height percentile (n = 412)**

Variables	ASWP		ASHP	
	Coefficient (95% CI)	p-value	Coefficient (95% CI)	p-value
Prematurity (ref = No)				0.03
Yes	-		−10.46 (−19.65, −1.28)	
Income quintiles (baht)		0.008		0.01
(ref = 30,001-40,000)				
40,001-500,000	−9.66 (−24.1, 4.75)		−9.80 (−22.8, 3.82)	
17,801-30,000	−1.68 (−15.2, 11.8)		−8.98 (−21.5, 3.55)	
10,001-17,800	−7.91 (−23.22, 7.39)		−15.1 (−29.1, −1.09)	
≤ 10,000	−20.1 (−34.0, −6.15)		−21.2 (−33.9, −8.40)	
Repeated GA (per time)	−1.28 (−2.36, −0.20)	0.02	−1.17 (−2.20, −0.14)	0.03
Choice of GA				0.02
(ref = GA only)	-			
GA combined RA/ PNB			−9.67 (−17.7, −1.63)	
Technique of GA				0.02
(ref = LMA)				
Facemask	-		−8.24 (−23.7, 7.2)	
ETT			−12.7 (−21.8, −3.59)	
Duration of GA (ref = <1)		0.26		0.74
1-3	9.09 (−1.98, 20.2)		3.69 (−8.42, 15.8)	
>3	5.81 (−10.1, 21.7)		6.54 (−10.05, 23.1)	
PD (ref = No-PRE)		0.005		0.11
at time of index GA				
Yes	−12.6 (−21.3, −3.76)		−6.84 (−15.2, 1.54)	
Time (ref = At index GA)		0.04		0.03
in no-PRE group				
At IQ test	5.41 (0.25, 10.56)		6.40 (0.68, 12.1)	
Interaction: time × PD	4.66 (−2.63, 11.95)	0.21	−1.65 (−9.74, 6.44)	0.69

p-value by F test statistic, Monthly income quintiles, 1 baht = US$ 0.033 at the time of data collection, ASWP; Age-specific weight percentile, ASHP; Age-specific height percentile, ETT; Endotracheal tube intubation, GA; General anesthesia, IQ; Intellligence quotient, LMA; Laryngeal mask airway, PD; Perioperative desaturation, PRE; Perioperative respiratory event, RA; Regional anesthesia, PNB; Peripheral nerve block, Time; Time of assessment.

## Discussion

Our study could not demonstrate any serious effect of transient perioperative desaturation on subsequent intelligence or on the change in age-specific weight and height percentile of children after non-cardiac surgery. According to the National Thai Survey in 2007, the average IQ score of southern Thai children (96.9) using the Standard Progressive Matrices (a nonverbal test) of IQ measurement (Raven 1938) was slightly lower than the IQ score in the no-PRE group (98.3) and higher than the IQ score in the PD group (94.7).

### PD and other confounders related to intelligence

In the current study, there was no significant impairment of intelligence assessed more than one year after developing transient hypoxic events (less than 60 seconds) during intraoperative or PACU period in non-chronic hypoxic children. Two previous studies of congenital cyanotic cardiac surgery have supported our finding in terms of no differences in neurodevelopment outcome of chronic hypoxia or cardiac arrest children when compared with healthy children or children with no cardiac arrest, respectively (Hövels-Gürich et al. 2001; Hansen et al. 2011). However, the reasons why PD itself was not associated with intelligence outcome in our study can be explained as follows. Firstly, desaturation in our study would have been for only a short period (less than 60 seconds) since our anesthesiologists attempted to reduce and correct PD immediately. Severe and long duration of PD may induce hypoxic ischemic brain injury and result in cell apoptosis and necrotic cell death (Baburamani et al. 2012). The lack of differences in IQ score in mild to moderate (SpO_2_ 86-94%) and severe desaturation (SpO_2_ ≤ 85%) compared to no-PRE (p = 0.27) in our study could also be due to the brief duration of PD. Secondly, intelligence in children is multifactorial. GA itself that includes anesthetic agents involving γ-aminobutyric acid receptors eg; inhalation agents, midazolam, and N-methyl-D-aspartate receptor antagonists such as ketamine, may affect neurodevelopment outcomes by disturbing mitochondrial morphogenesis and resulting in neuroapoptosis in the developing rat brain (Boscolo et al. 2013) although evidence in human babies is still uncertain (Sananes et al. 2012; Sun 2010). Our study could not identify any significant association between various types of anesthetic agents and IQ score. However, our finding that the number of repeated episodes of GA was not significantly different between the two groups (p = 0.08) but was a significant factor related to lower IQ (p = 0.01) suggests that GA itself rather than PD may influence neurodevelopmental outcomes in preschool children undergoing non-cardiac surgery. Our result was comparable to a study by Kayaalp et al. (Kayaalp et al. 2006) which found that repeated GA may influence psychological health in children.

Other factors reported to have an impact on intelligence outcome are family characteristics. Our study showed that low mother’s education (p < 0.001), low father’s education (p = 0.007) and no regular training by parents (p = 0.007) were significant predictors for low IQ. Our findings were consistent with a study by Vanderveen et al. (Vanderveen et al. 2009) showing that high parent education and early intervention in premature infants such as infant stimulation improved neurodevelopment outcomes in children aged less than 36 months.

### PD and association with PDD

In the current study, there was no evidence that PD was associated with PDD (p = 1). A meta-analysis showed some perinatal and neonatal factors such as fetal distress, birth injury or trauma, multiple birth, maternal hemorrhage, low birth weight or low 5-minute apgar score, were associated with autism, whereas delivery by anesthesia was not a risk factor for autism (Gardener et al. 2011). However, no previous studies have shown an association between receiving GA or developing PD and having PDD in preschool children.

### PD and other confounders related to change in age-specific weight and height percentile

In the current study, desaturation occurring intraoperatively or at PACU in preschool children was not significantly associated with subsequent change in age-specific weight or height percentile examined between 2 times: at the time of index GA and at the time of IQ test. Multivariate mixed effects regression was performed to estimate the change in age-specific weight and height percentile from the values at the time of index GA since we found a significant association between PD and lower ASWP at the time of index GA (p = 0.007). Our study showed that the number of repeated episodes of GA was significantly associated not only with lower IQ but also with a lower age-specific weight and height percentile (p = 0.02 and p = 0.03, respectively). Moreover, anesthesia-related factors associated with lower age-specific height percentile were GA combined with regional anesthesia and technique of GA with ETT. According to our cohort study, it may be more likely that low age-specific weight or height percentile from poor nutrition was a determinant for repeated surgery, repeated GA and using a particular choice and technique of GA. However, to date, this has not been shown in other relevant studies.

Other confounders related to lower age-specific weight and/or height percentile from the time of index GA to the time of IQ test found in our study were not surprising: low monthly family income (≤ 10,000 baht or US$ 333.33) (p = 0.008 for ASWP and p = 0.01 for ASHP) and prematurity (p = 0.03). Moilanen et al. (Moilanen et al. 2009) reported that family poverty was associated with slower growth in early childhood. The association between low income and lower age-specific weight and height percentile in our study could possibly be explained by poor nutritional status, which is an important factor for intelligence and growth (Sandjaja et al. 2013) but nutritional status was not assessed in our study. Bocca-Tjeertes et al. (Bocca-Tjeertes et al. 2011) also supported our finding that growth restraint was associated with preterm-born children compared to term-born children.

### Strengths and limitations

The strength of the current study was that children in the two groups were matched on sex, age, year of having index GA, type of surgery and choice of anesthesia to reduce the level of confounding between the PD and no-PRE groups. In addition, this study employed multiple linear regression analysis which takes into account adjustment for other confounding factors. Moreover, the intelligence outcome assessment was carried out by only one experienced pediatric psychometrist, who was not aware of the child’s exposure status (PD or no-PRE) at the time of the assessment.

There were some limitations of the study. Firstly, assessment of the outcomes was done only once. We did not assess intelligence in presumed mentally healthy children before children received GA. We could not know if any children had mildly delayed milestones at the index GA, which may have resulted in a lower IQ score than normal milestone children. Secondly, the newer version of the Standford Binet (the Standford Binet 5th edition) test was not performed in our study because Songklanagarind Hospital provides only the performance subtest (nonverbal IQ subtest) for the Standford Binet 5th edition while the verbal subtest of SB5 has not yet been standardized in Thai language. We believe the Standford Binet form L-M test would provide more benefit to school children and parents in terms of neurodevelopment than the performance subtest of the Standford Binet 5th edition. Lastly, because our study was based on an effect size of 0.5 (6/12), the sample size was too small to detect a smaller difference in IQ scores between the two groups. Furthermore, the variability in IQ (in each group) was actually greater than SD of 12 (SD of 16 and 17.5 in the PD and no-PRE group, respectively).

Based on the study design and method of analysis, the validity of our findings is plausible. Because the study was done in a referral hospital, the generalizability to southern Thai children population is probably satisfactory.

## Conclusions

So far, there is no evidence that brief PD in this study had any serious or significant growth and development consequence to the child. Longer-term follow up on a larger sample size may be needed.

## Electronic supplementary material

Additional file 1: **Definition of training by parents.** (PDF 32 KB)

Additional file 2: **Definition of child’s capability.** (PDF 86 KB)

## Abbreviations

PD: Perioperative desaturation
PDD: Pervasive developmental disorders
PRE: Perioperative respiratory events
GA: General anesthesia
ASA: American Society of Anesthesiologists
PACU: Post-anesthetic care unit
IQ: Intelligence quotient
ASWP: Age-specific weight percentile
ASHP: Age-specific height percentile
SpO2: Oxygen saturation by pulse oximetry.
