# Supplementary material for: Comparison of intelligence, weight and height in children after general anesthesia with and without perioperative desaturation in non-cardiac surgery: a historical and concurrent follow-up study
Source: Springerplus. 2014 Mar 29;3:164. doi: 10.1186/2193-1801-3-164 (PMC4320222; doi:10.1186/2193-1801-3-164)
Supplement: Supplementary file 1 — Additional file 1: Definition of training by parents. (PDF 32 KB) [file 40064_2014_1473_MOESM1_ESM.pdf]

Additional file 1

Definition of training by parents

Training by parents was defined as developmental stimulation and training by parents or caregivers according to the following criteria; (1) teaching the child to do things by him/herself compatible with age: if this was done regularly, it was considered to be regular stimulation and training; if this was done once in a while, it was considered to be partial stimulation and training; (2) asking their child simple questions to stimulate his/her mind: if this was done regularly, it was considered to be regular stimulation and training; if this was done once in a while, it was considered to be partial stimulation and training; (3) taking their child outside for a ride or to the playground and stimulating the child by showing things, people or animals: if this was done regularly by both taking them outside and stimulating the mind, it was considered to be regular stimulation and training; if this was done once in a while, it was considered to be partial stimulation and training; if the child was taken outside but the parents/caregivers did not stimulate the child's mind, it was considered to be partial stimulation and training; (4) buying their child a stimulating toy and playing with him/her: if the parents bought toys and played with them regularly, it was considered to be regular stimulation and training; if the parents bought the child toys but did not play with them, it was considered to be partial stimulation and training. If none of the 4 items was performed, it was considered to be no stimulation or training. If at least 2 out of 4 criteria were identified as regular stimulation and training, it was considered to be regular stimulation and training; otherwise, it was considered to be partial stimulation and training. Training and stimulation by parents was classified into 3 categories namely; none, partial or regular.
