# Supplementary material for: Comparison of intelligence, weight and height in children after general anesthesia with and without perioperative desaturation in non-cardiac surgery: a historical and concurrent follow-up study
Source: Springerplus. 2014 Mar 29;3:164. doi: 10.1186/2193-1801-3-164 (PMC4320222; doi:10.1186/2193-1801-3-164)
Supplement: Supplementary file 2 — Additional file 2: Definition of child’s capability. (PDF 86 KB) [file 40064_2014_1473_MOESM2_ESM.pdf]

Additional file 2

Definition of child’s capability

Children’s capability was defined as the ability to do things by themselves compatible with their ages. For children 2 to 3 years of age, ability to climb up and down stairs, climb off their bed and take off their clothes. For children 3 to 5 years of age, ability to eat with a spoon, take a shower, brush their teeth and ride a 3-wheeled bicycle. For children 5 to 6 years of age, ability to get dressed in clothes with no buttons, go to the toilet and wear shoes without shoelaces. For children 6 to 8 years of age, ability to draw and write on paper, get dressed and do up buttons and tie shoelaces. If children required full help for all activities compatible with their age, they were considered to be requiring full help. If children were able to do all activities without any help compatible with their age, they were considered to be requiring no help; otherwise, they were considered to be requiring partial help. Children’s capability was classified into 3 categories, namely requiring full help, partial help or no help.
